# Cross-Regional Comparison of Type 2 Inflammatory Markers in Chronic Rhinosinusitis With Nasal Polyposis: Insights From the Gulf Region and Canada

**DOI:** 10.7759/cureus.87500

**Published:** 2025-07-08

**Authors:** Ali A Al-Fraihat, Abdullah I Malek, Audrey Pelletier, Leandra Endam, Martin Desrosiers, Iyad Hamadi

**Affiliations:** 1 Department of Otolaryngology - Head and Neck Surgery, Dubai Health - MBRU, Dubai, ARE; 2 Research Center, University of Montreal Hospital Center (CRCHUM), Montreal, CAN; 3 Department of Otolaryngology - Head and Neck Surgery and Allergy, McGill University, Montreal, CAN

**Keywords:** chronic rhinosinusitis, crswnp, diversity and inclusion, eosinophils, nasal polyps, type 2 inflammation

## Abstract

Objective

Chronic rhinosinusitis with nasal polyposis (CRSwNP) is prevalent worldwide, but regional variations in disease endotypes have been described. The Gulf region is unique in genetic background and environmental conditions, potentially influencing disease characteristics. This study aimed to compare phenotypic traits and serum biomarkers associated with type 2 disease between CRSwNP patients in the Gulf and Canada.

Study design

A retrospective observational study was conducted in a tertiary center in Dubai, comparing newly diagnosed CRSwNP patients from the United Arab Emirates (UAE), representative of the UAE, with two Canadian cohorts: severe chronic rhinosinusitis (CRS) patients, (Genetics of Chronic Rhinosinusitis 1 (GCRS1)) and CRSwNP patients (Genetics of Chronic Rhinosinusitis 2 (GCRS2)).

Methods

Serum eosinophilia, white blood cell (WBC), and immunoglobulin E (IgE) levels were analyzed to identify potential differences or similarities between the populations.

Results

Serum eosinophil counts and the percentage of subjects with high serum eosinophilia values (≥300 cells/µL) were similar across the UAE and Canadian groups. However, total serum IgE levels were higher in the UAE cohort, while reported allergy rates were significantly lower compared to the Canadian groups. Type 2 comorbidities were more frequently reported in the Canadian cohorts, potentially reflecting differences in diagnostic practices or patient reporting.

Conclusion

Despite environmental and population differences, the immunological profile of CRSwNP disease in the Gulf region closely mirrors that in North America, suggesting that management strategies developed and used in Western countries are applicable in the Gulf. Eosinophil screening in CRSwNP patients remains valuable to detect elevated levels, potentially indicative of vasculitis.

## Introduction

Chronic rhinosinusitis (CRS) is a heterogeneous disease caused by local inflammation of the sinonasal mucosa and paranasal sinuses, significantly impacting patients’ social, physical, and economic well-being [1]. This condition has a global reach, with prevalence rates varying across regions: 12% in the United States of America, 4.6% in Canada, 10.9% in Europe, and 8% in China [2,3]. However, a notable gap exists in our understanding of CRS with nasal polyps (CRSwNP) in the Gulf region (Saudi Arabia, United Arab Emirates (UAE), Kuwait, Oman, Bahrain, and Qatar), an area home to 59.6 million people living in an environment distinct from European and Asian populations [4].

The Gulf region’s unique genetic background and environmental conditions suggest potential differences in disease manifestation and therapeutic needs compared to the Americas, Asia, and Europe. While Asian studies indicate a lower prevalence of type 2 disease, anecdotal evidence from the Gulf area hints at severe, recalcitrant CRSwNP, possibly signifying an overrepresentation of type 2 disease. However, this remains speculative due to the lack of specific data on CRSwNP in the Gulf region, potentially hindering the delivery of appropriate care.

Recent advances in CRS management have led to a “precision medicine” approach, where therapy is tailored based on patient-specific features indicative of the underlying disease process. Historically, this approach relied on CRS phenotypes, defined by the presence or absence of hyperplastic changes of the sinus mucosa protruding into the nasal cavity, or nasal polyps (NP). However, emerging evidence suggests that both CRSwNP and CRS without NP (CRSsNP) exhibit heterogeneous pathogenic mechanisms [5]. Consequently, the focus had shifted toward identifying characteristics that reveal disease mechanisms amenable to targeted therapy, leading to the concept of inflammatory pattern, or “endotype” [6].

Inflammatory endotypes in CRS are broadly categorized into type 2 and non-type 2 disease patterns, distinguished by their inflammatory cell populations and cytokine profiles. Type 2 inflammatory responses are characterized by elevated eosinophilia and increased levels of type 2 inflammatory cytokines such as interleukin (IL)-4, IL-5, IL-13, and transforming growth factor-beta (TGF-β) [7]. In contrast, non-type 2 inflammatory responses are typically neutrophil-mediated and associated with higher levels of interferon‐gamma (IFN‐γ) and IL-17 [8]. The presence of a type 2 component can be approximated by using serum biomarkers such as eosinophilia (≥300 cells/µL) or total serum immunoglobulin E (IgE) level (>100 kU/L) [9]. Additionally, certain phenotypic traits such as asthma, allergic rhinitis, and eczema can suggest an underlying type 2 disorder in CRSwNP patients.

While type 2 disease predominates in European and North American CRSwNP populations, its prevalence may vary in other geographic areas. Environmental differences, including bacterial and viral burdens, coupled with genetic variations adapted to local conditions, may lead to distinct mechanisms of disease development. This is exemplified by the increased proportion of non-type 2 CRSwNP reported in Asian populations [10,11]. As the Gulf represents a unique geographical area, potential variations in disease patterns could impact endotype-based approaches to CRS and strategies for prescribing biologic medications targeting type 2 inflammation.

Given these considerations, we aimed to explore differences in the prevalence of type 2 CRS disease in the Gulf population. Our study compares the prevalence of asthma, allergic rhinitis, and eczema, as well as levels of serum eosinophilia, white blood cells (WBCs), and IgE, between a United Arab Emirates (UAE) CRSwNP population and a similar CRS population in Canada. This comparison will provide valuable insights into the potential uniqueness of CRSwNP presentation in the Gulf region and inform appropriate management strategies.

## Materials and methods

Study design

This comparative retrospective observational study assessed serum eosinophilia levels in newly diagnosed CRSwNP patients from a tertiary center in Dubai, representative of the UAE population, and compared them to two Canadian cohorts. The primary aim was to elucidate potential similarities or differences in serum eosinophilia, white blood cell counts, and total IgE levels between these populations.

This study was approved by the Institutional Review Board (MBRU IRB-2024-248) and conducted in accordance with the relevant guidelines and regulations and the Declaration of Helsinki.

Study populations

UAE Population

A representative random sample (n=83) of adult patients (≥18 years) diagnosed with CRSwNP at the otolaryngology department of a Dubai hospital was extracted from the electronic medical records (EMR) at a tertiary center in the UAE. The presence of nasal polyposis was also confirmed and documented using nasal endoscopy. Exclusion criteria included any pediatric patients, patients who did not meet the guidelines stated above, and patients without CT scans.

Canadian Population

Two Canadian cohorts were analyzed: (1) GCRS1 severe CRS population (n=203), which prospectively recruited subjects with severe CRS defined as either persistent symptoms despite previous endoscopic sinus surgery (ESS) [12] or a history of multiple ESS procedures for CRS, regardless of the outcome, and (2) GCRS2 “real-world” CRSwNP population (n=468), consisting of subjects recruited for the diagnosis of CRSwNP, regardless of disease extent, prior therapy, or treatment response [13].

This study was approved by the McGill University Health Centre Institutional Review Board (GCRS1: ND05.082, GCRS2: ND06.054) and the Centre Hospitalier de l’Université de Montréal. Informed consent was obtained from all participants. CRS was defined according to the 2004 American Academy of Otolaryngology-Head and Neck Surgery guidelines [7].

Data collection

All data collected from patients were stored anonymously on a data collection tool, with each patient assigned a patient number. Data was stored in a secure location, and no transmission of data was done that could lead to loss of confidentiality or patient identifiers. For the UAE group, IgE levels were sometimes taken from previous blood test results, ranging from six months to seven years before the study.

Statistical analyses

All statistical analyses were performed using GraphPad Prism version 10.3.1 (GraphPad Software, San Diego, CA). The study compared data across three groups: GCRS1, GCRS2, and UAE.

Demographic Analysis

Demographic characteristics were analyzed for GCRS1 (n=203), GCRS2 (n=468), and UAE (n=83). Continuous variables (e.g., age) were presented as mean ± standard deviation and analyzed using one-way analysis of variance (ANOVA). Categorical variables (gender, asthma status, smoking status, allergies, acetylsalicylic acid (ASA) sensitivity, and eczema) were presented as counts and percentages and analyzed using Fisher’s exact test. To account for multiple comparisons, a false discovery rate (FDR) correction was applied, and both p-values and q-values were reported. Results were considered statistically significant if both p-value and q-value were less than 0.05.

Immunological Profile Analysis

Eosinophil counts and percentages and white blood cell (WBC) counts were compared across the three groups (GCRS1: n=203, GCRS2: n=468, and UAE: n=83). Total serum IgE levels (KU/L) were analyzed for GCRS1 (n=201), GCRS2 (n=440), and UAE (n=36). One-way analysis of variance (ANOVA) was performed to assess differences between groups, and post hoc analysis was carried out using Tukey’s multiple comparisons tests, as well as the correction for multiple comparisons by controlling the FDR using the two-stage linear step-up procedure of Benjamini, Krieger, and Yekutieli. Data were visualized using Tukey box-and-whisker plots with individual data points overlaid. Descriptive statistics included mean, standard deviation, standard error of the mean, and coefficient of variation. The statistical significance threshold was set at p<0.05 for all analyses.

## Results

Demographic characteristics

Our study included newly diagnosed CRSwNP patients from the UAE (n=83) and two Canadian cohorts (GCRS1 (n=203) and GCRS2 (n=468)). GCRS1 comprised patients with severe CRSwNP, defined as persistent symptoms post-ESS or a history of multiple ESS procedures. GCRS2 included patients with any CRSwNP phenotype, regardless of disease extent, prior therapy, or treatment response.

Demographic analysis revealed significant differences in age and type 2 disease-associated phenotypic traits among the populations. The UAE cohort was notably younger than both Canadian groups (mean age: UAE, 43.14 years; GCRS1, 52.23 years; GCRS2, 49.69 years; p<0.0001). Self-reported diagnoses of type 2 comorbidities were less frequent in the UAE than in the Canadian populations, including asthma (UAE: 27.7%, GCRS1: 53.7%, GCRS2: 53.4%; p<0.0001), allergies (UAE: 25.3%, GCRS1: 60.5%, GCRS2: 66.0%; p<0.0001), ASA intolerance (UAE: 0.0%, GCRS1: 28.6%, GCRS2: 27.1%; p<0.0001), and eczema (UAE: 2.4%, GCRS1: 20.2%, GCRS2: 17.7%; p<0.0001). Table 1 summarizes demographic data.

**Table 1:** Demographic data ^1^Mean ± standard deviation ^2^n/N (%) ^3^One-way ANOVA, Fisher’s exact test for categorical variables ^4^False discovery rate correction for multiple testing ANOVA: analysis of variance, UAE: United Arab Emirates, GCRS1: Genetics of Chronic Rhinosinusitis 1, GCRS2: Genetics of Chronic Rhinosinusitis 2, ASA: acetylsalicylic acid

Characteristic	GCRS1	GCRS2	UAE	p-value^3^	q-value^4^
	(N=203)	(N=468)	(N=83)		
Age (years)^1^	52.23±12.86	49.69±12.56	43.14±13.11	<0.0001	<0.0001
Gender^2^				0.2094	0.6383
Male	105/203 (51.7%)	275/468 (58.7%)	44/83 (53.0%)		
Female	98/203 (48.3%)	194/468 (41.4%)	39/83 (47.0%)		
Asthma^2^				<0.0001	<0.0001
Yes	109/203 (53.7%)	250/468 (53.4%)	23/83 (27.7%)		
No	74/203 (46.3%)	218/468 (46.6%)	60/83 (72.3%)		
Smoking^2^	22/203 (10.8%)	46/468 (9.8%)	11/83 (13.3%)	0.5804	0.5258
Allergies^2^	134/203 (66.0%)	283/468 (60.5%)	21/83 (25.3%)	<0.0001	<0.0001
ASA^2^	58/203 (28.6%)	127/468 (27.1%)	0/83 (0.0%)	<0.0001	<0.0001
Eczema^2^	41/203 (20.2%)	83/468 (17.7%)	2/83 (2.4%)	<0.0001	<0.0001

Serum eosinophilia and white blood cell counts

**Figure 1:**
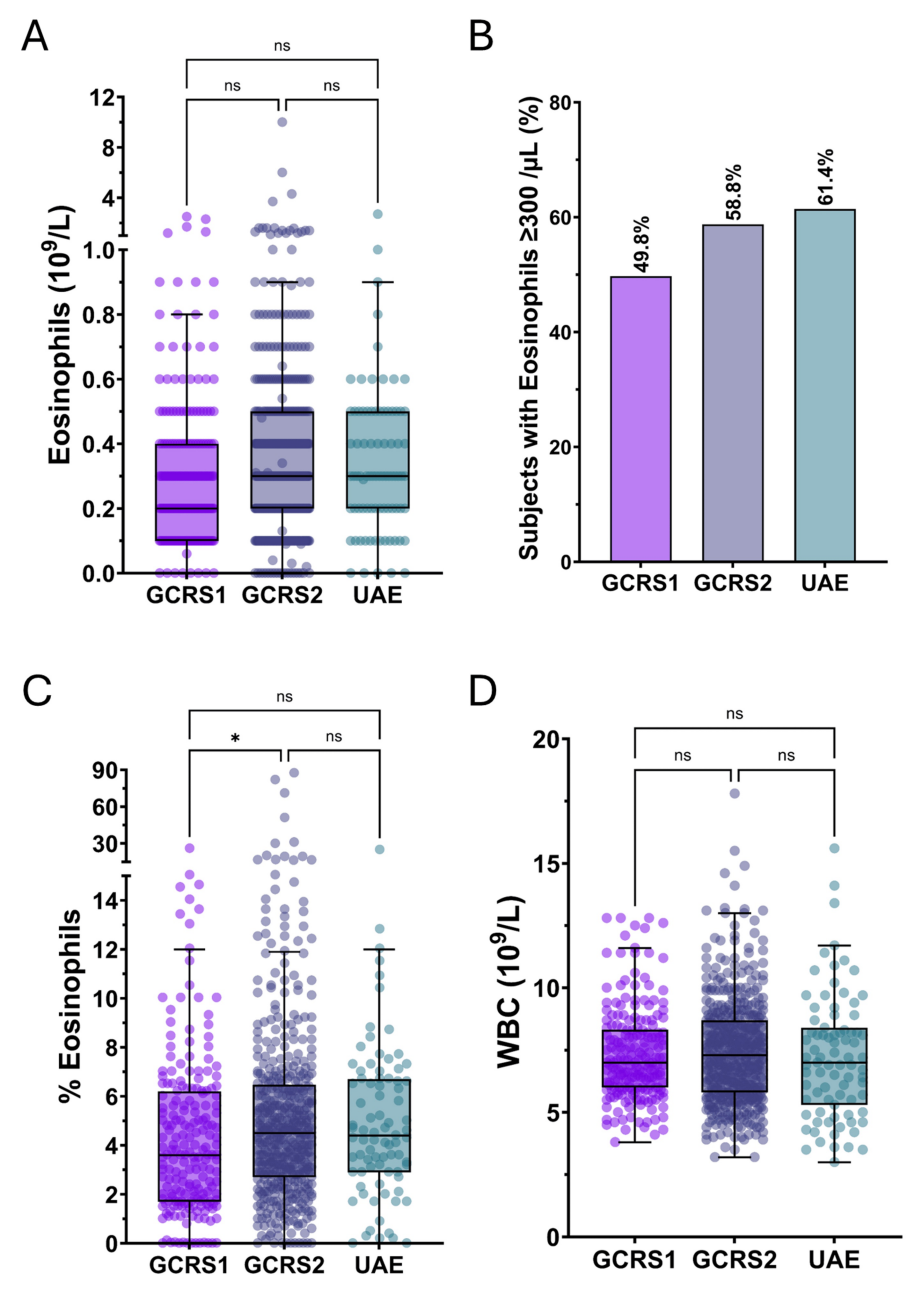
Comparison of serum eosinophilia and WBC levels in the CRSwNP cohort from the Gulf (UAE) with the Canadian GCRS1 and GCRS2 CRSwNP population Statistical significance is indicated by asterisks (*p≤0.05). CRSwNP: chronic rhinosinusitis with nasal polyposis, ns: non-significant, WBC: white blood cell, UAE: United Arab Emirates, GCRS1: Genetics of Chronic Rhinosinusitis 1, GCRS2: Genetics of Chronic Rhinosinusitis 2

Comparison of the serum eosinophilia and WBC levels in the CRSwNP cohort from the Gulf (UAE) with the Canadian GCRS1 and GCRS2 CRSwNP population is shown in Figure 1.

Data are presented as Tukey box plots with superimposed individual values as shown in Figures 1A, 1C, 1D. A comparison of the distribution of serum eosinophilia levels as depicted in Figure 1A revealed similar patterns across all three cohorts. The mean eosinophil counts were 358±331 cells/µL in the UAE cohort, 327±317 cells/µL in the GCRS1 group, and 406±633 cells/µL in the GCRS2 group. These differences were not statistically significant (p=0.2022). A limited number of high values was noted, which would currently prompt assessment to exclude underlying conditions such as vasculitis. The proportion of subjects with serum eosinophilia ≥300 cells/µL shows a high frequency of eosinophil-defined type 2 endotype in CRSwNP patients in all three populations, as shown in Figure 1B, with 61% of the UAE population, 50% of the GCRS1 group, and 59% of the GCRS2 group. These differences were not statistically significant (p=0.0653).

The percentage of eosinophils (%EOS) among total white blood cells (WBCs) was assessed across the three cohorts as depicted in Figure 1C. The mean %EOS was 5.0±3.5% in the UAE cohort, 4.4±3.5% in the GCRS1 group, and 5.6±7.3% in the GCRS2 group. A statistically significant difference was observed between the GCRS1 and GCRS2 groups (p=0.0425), with the GCRS2 group showing a higher eosinophil percentage, although this increase was accompanied by substantial variability. No significant difference was observed between the UAE cohort and the Canadian populations.

Total WBC counts were also compared across the three groups, as shown in Figure 1D. The mean WBC count was 7.2±2.5×10⁹/L in the UAE cohort, 7.3±1.9×10⁹/L in the GCRS1 group, and 7.4±2.1×10⁹/L in the GCRS2 group. These differences were not statistically significant (p=0.6205), indicating a similar overall WBC distribution among the groups. Therefore, the observed difference in the %EOS between the two Canadian groups, despite no significant change in absolute eosinophil counts or total WBC counts, could be due to variations in the distribution of other WBC subtypes.

Total serum IgE

**Figure 2:**
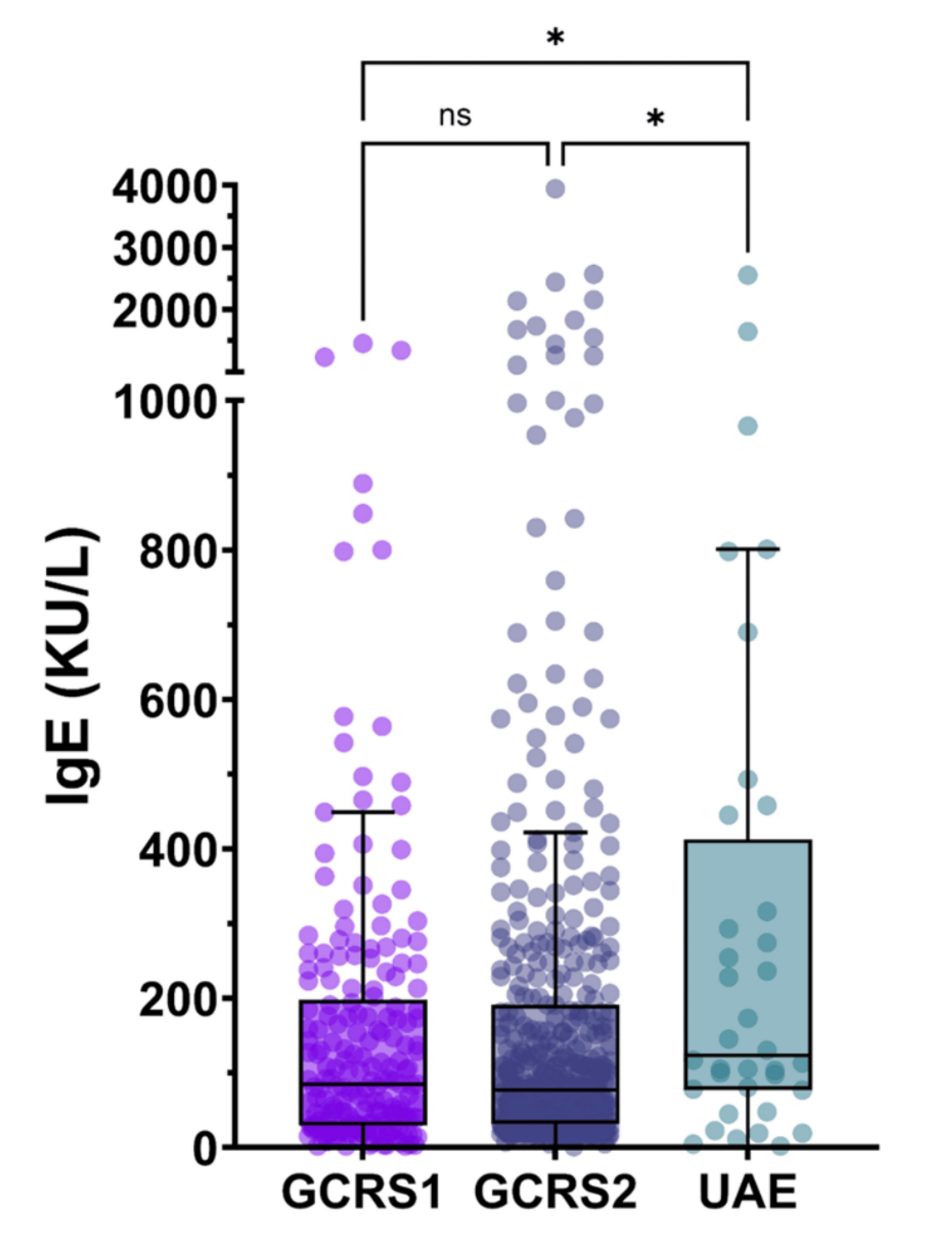
Comparison of total serum IgE levels in UAE and Canadian CRSwNP populations shown as Tukey box plots with superimposed individual values Statistical significance is indicated by asterisks (*p≤0.05, **p≤0.01). CRSwNP: chronic rhinosinusitis with nasal polyposis, ns: non-significant, UAE: United Arab Emirates, GCRS1: Genetics of Chronic Rhinosinusitis 1, GCRS2: Genetics of Chronic Rhinosinusitis 2

Total serum IgE levels were available for a subset of patients (UAE: n=36, GCRS1: n=201, GCRS2: n=440). Figure 2 illustrates that despite the limited sample size in the UAE cohort, IgE levels showed statistically significant differences among the groups.

The UAE cohort exhibited higher mean IgE levels (334.6±510.9 KU/L) compared to GCRS1 (157.3±216.1 KU/L) and GCRS2 (193.0±371.4 KU/L) (p=0.0163). However, given the large standard deviations and the small UAE sample size for total serum IgE levels, these results should be interpreted with caution.

## Discussion

This study presents a preliminary assessment of the prevalence of type 2 inflammation in CRSwNP patients from the Gulf region, focusing on phenotypic traits and biomarkers such as serum eosinophilia and total serum IgE. These biomarkers serve as a surrogate for more direct measurements from biopsy samples.

Our findings suggest that the prevalence of type 2 biomarkers in CRSwNP patients in the UAE is comparable to that of the North American CRSwNP patients. Despite potential differences in environmental, social, and genetic factors that might influence CRS development in the Gulf, serum biomarker data support a similar underlying disease mechanism across populations. This conclusion aligns with prior findings in the region, such as a study from Kuwait that reported 41.7% of adult asthmatics had eosinophil counts ≥ 300 cells/µL [14].

While all groups showed elevated IgE levels, a notable difference was seen between the populations. The UAE cohort had markedly higher IgE levels compared to both Canadian cohorts, although this finding should be interpreted cautiously due to the smaller sample size in the UAE group. The elevated IgE levels in the Gulf population may suggest a heightened allergic component in the UAE cohort or potentially indicate an underlying inborn error of immunity, as noted in prior research [15]. Further investigation is required to clarify the reasons behind this disparity. Nevertheless, the higher IgE levels in the UAE cohort highlight the potential influence of distinct factors that may affect the clinical management of CRSwNP in this region.

A notable discrepancy exists between total serum IgE levels and self-reported allergy rates in the UAE cohort. Despite higher IgE levels, self-reported allergies were significantly lower, suggesting potential underreporting or underdiagnosis, potentially due to cultural or healthcare system differences. Environmental factors unique to the region, such as exposure to dust and pollution, may elevate IgE levels without manifesting typical allergic symptoms. This raises the hypothesis that elevated IgE levels in the UAE may reflect an underlying allergic or immune response not fully captured by self-reported data or conventional diagnostic criteria used in different regions. Further research, including allergen profiling and more systematic allergy testing in the Gulf, is needed to clarify this apparent disconnect between biomarkers and reported allergies.

Moreover, demographic differences were observed, particularly in age and the frequency of phenotypic traits associated with type 2 inflammation. UAE patients were younger than their Canadian counterparts, which could reflect differences in healthcare access or potentially indicate an earlier onset of disease in the Gulf. Early onset of disease may suggest a genetic predisposition to CRSwNP in this population.

The lower prevalence of self-reported type 2 comorbidities, such as asthma, in the UAE cohort compared to the Canadian patients may be attributed to underreporting by both patients and healthcare providers. Given that the general population’s asthma prevalence is similar in the Gulf and Canada [16,17], it is plausible that the actual rates of asthma among UAE CRSwNP patients are under-captured. This could reflect differences in the thoroughness of clinical assessments, with structured questionnaires in the Canadian Genetics of Chronic Sinusitis Study likely providing more accurate data on comorbidities.

Serum eosinophilia, a reflection of airway inflammation, is a key marker of type 2 disease. It has well-documented implications for medication response in asthma [18] and can also influence adverse events in both asthma and CRSwNP [19]. Since eosinophilia screening has not been universally adopted into clinical practice, these findings offer reassurance that Gulf patients can likely be managed according to European guidelines. In fact, despite regional differences, CRS patients from the UAE and North America show remarkable similarity in key biomarkers associated with CRS disease. In this context, educational efforts should focus on raising awareness about identifying type 2 comorbidities in CRSwNP patients.

It is important to recognize that this study represents an early, isolated report, and these findings need to be replicated in larger populations. Further studies should also include biopsy-based investigations to confirm the type and extent of inflammatory cell infiltration, alongside research into Gulf-specific etiologies, risk factors, pathogens, and potential genetic variations that may contribute to disease development and persistence.

Additionally, isolated cases of high baseline eosinophilia in a few patients raise concerns. Such elevated levels have been previously described [20], and current guidelines mandate that these patients should undergo serological testing to rule out conditions such as vasculitis. Implementing routine eosinophilia screening in clinical practice should be considered to detect any underlying conditions early and enhance the management of these patients.

Study limitations

This study has several limitations. First, the sample size of the UAE cohort, particularly for total serum IgE measurements, was relatively small, which may limit the generalizability of findings and increase variability. Second, differences in data collection methods between the UAE and Canadian cohorts, such as structured questionnaires in Canada versus EMR review in the UAE, may have influenced the reporting of comorbidities. Finally, biopsy confirmation of type 2 inflammation was not performed, which could have better full captured underlying tissue inflammation.

## Conclusions

This study represents the first comparative analysis of type 2 inflammation biomarkers in CRSwNP patients from the Gulf region and North America. Despite environmental, genetic, and healthcare system differences, patients in the UAE and Canada showed similar levels of serum eosinophilia and total IgE, supporting the applicability of current European CRSwNP management guidelines in the Gulf region. The discrepancy between higher IgE levels and lower self-reported allergy rates in the UAE cohort suggests potential underdiagnosis of type 2 comorbidities, emphasizing the need for enhanced diagnostic awareness. These findings underscore the importance of recognizing universal inflammatory patterns in CRSwNP while encouraging further region-specific research.
